# Preceding infection and risk of stroke: An old concept revived by the COVID-19 pandemic

**DOI:** 10.1177/1747493020943815

**Published:** 2020-07-24

**Authors:** Kieron South, Laura McCulloch, Barry W McColl, Mitchell SV Elkind, Stuart M Allan, Craig J Smith

**Affiliations:** 1 Division of Neuroscience and Experimental Psychology, Lydia Becker Institute of Immunology and Inflammation, School of Biological Sciences, Faculty of Biology, Medicine and Health, 5292 The University of Manchester, Manchester Academic Health Science Centre, Manchester, UK; 2 Centre for Discovery Brain Sciences, UK Dementia Research Institute, University of Edinburgh, Edinburgh, UK; 3 Vagelos College of Physicians and Surgeons and Mailman School of Public Health, 5798 Columbia University, New York, NY, USA; 4 Division of Cardiovascular Sciences, Lydia Becker Institute of Immunology and Inflammation, School of Biological Sciences, Faculty of Biology, Medicine and Health, 5292 The University of Manchester, Manchester Academic Health Science Centre, Manchester, UK; 5 Manchester Centre for Clinical Neurosciences, Manchester Academic Health Science Centre, Salford Royal NHS Foundation Trust, Salford, UK

**Keywords:** COVID-19, infection, ischemic stroke, risk factors, SARS-CoV-2, stroke prevalence, vascular events

## Abstract

Anecdotal reports and clinical observations have recently emerged suggesting a relationship between COVID-19 disease and stroke, highlighting the possibility that infected individuals may be more susceptible to cerebrovascular events. In this review we draw on emerging studies of the current pandemic and data from earlier, viral epidemics, to describe possible mechanisms by which SARS-CoV-2 may influence the prevalence of stroke, with a focus on the thromboinflammatory pathways, which may be perturbed. Some of these potential mechanisms are not novel but are, in fact, long-standing hypotheses linking stroke with preceding infection that are yet to be confirmed. The current pandemic may present a renewed opportunity to better understand the relationship between infection and stroke and possible underlying mechanisms.

## Introduction

### The SARS-CoV-2 global pandemic

At the time of writing, the global number of confirmed severe acute respiratory syndrome coronavirus 2 (SARS-CoV-2) cases is approaching 9 million, with over 470,000 reported fatalities. The current novel coronavirus outbreak began to receive worldwide media attention in early January 2020 with the earliest cluster of cases traced back to December 2019 in the city of Wuhan in China. By 30 January the World Health Organization (WHO) declared the outbreak a “public health emergency of international concern” and, after cases were reported in 210 countries, the outbreak was recognized by WHO as a pandemic on 11 March 2020.

SARS-CoV-2 is a member of the *betacoronavirus* genus of the *coronaviridae* family of enveloped, single-stranded RNA viruses, several of which are known to cause mild respiratory disease in humans. It was named because of its similarity to SARS-CoV, the virus responsible for an epidemic in 2002–2003 that infected approximately 8000 people with almost 800 fatalities. Both SARS-CoV and SARS-CoV-2 cause acute respiratory symptoms but due to enhanced rates of transmission derived from transmission from asymptomatic individuals^1^ and a high level of early viral shedding in the upper respiratory tract,^2^ this recent pandemic has attained a large global impact.

Angiotensin-converting enzyme 2 (ACE2), the “receptor” for host cell entry of SARS-CoV-2,^3^ is most prominently expressed on the surface of lung alveolar epithelial cells, venous and arterial endothelial cells, arterial smooth muscle cells and enterocytes of the small intestine.^4^ Notably, considering the possible neurotropism of SARS-CoV-2 discussed later, ACE2 is also found on cardio-respiratory neurons of the brainstem, in the hypothalamus and the motor cortex.^5^ There is evidence that SARS-CoV (and possibly SARS-CoV-2) is able to infect lymphocytes, monocytes and lymphoid tissues.^6^ This tissue distribution is a critical determinant of the COVID-19 disease course and may drive some of the thromboinflammatory alterations that might influence stroke pathophysiology, as discussed in this review.

### Clinical presentation of COVID-19

Unlike its predecessor SARS, COVID-19 manifests as a broad spectrum of disease severity from a completely asymptomatic state of infection, through mild flu-like symptoms, to the life-threatening acute respiratory distress syndrome (ARDS). It has been estimated that as many as 86% of cases in China were asymptomatic or mildly symptomatic and were, therefore, undocumented.^1^ The multitude of factors contributing to this disparity in disease severity are not yet fully understood and are likely to include genetic, environmental and host response factors and would, therefore, be outside the scope of this review. However, it is already clear that disease progression is, to some extent, linked to viral load,^7^ age, sex, ethnicity and comorbidity.^8,9^

At onset of illness the most common symptoms are fever, cough, myalgia, anosmia and fatigue. Common chest radiological findings are bilateral ground–glass opacity, interlobular septal thickening, and thickening of the pleura.^10^ In patients who went on to develop ARDS, pleural effusion, lymphadenopathy and round cystic changes were also observed, similar to those seen previously in SARS,^11^ Middle East respiratory Syndrome (MERS)^12^ and H_5_N_1_ influenza.^13^ Patients who develop ARDS experience severe hypoxemia, and the leading causes of mortality are respiratory failure, heart failure, fulminant myocarditis and multiple organ failure.

### Cerebrovascular complications in COVID-19 patients

The possible relationship between respiratory tract infection and the incidence of stroke, particularly ischemic stroke, is not a new concept. Early case–control studies identified respiratory tract infections as a significant risk factor across all age groups despite adjusting for other known vascular risk factors.^14^ A large case-series analysis of UK medical records identified a significant risk of either first stroke or recurrent stroke associated with a diagnosis of acute respiratory tract infection.^15^ This risk was highest in the first few days after infection, steadily declining thereafter but remaining elevated over baseline for some time. The incidence ratio of first stroke was found to be 3.19 (95% CI 2.81 to 3.62) within three days of infection and 2.09 (95% CI 1.89 to 2.32) within 14 days. A later retrospective case-crossover study of administrative data in the US, focusing on respiratory tract infections defined using Centers for Disease Control and Prevention criteria as “influenza-like illness”, identified a similar risk of ischemic stroke within 15 days of infection (odds ratio 2.88, 95% CI 1.86 to 4.47).^16^

Large, systematically collated datasets are not yet available for the current SARS-CoV-2 pandemic and, as such reliable estimates of the associated risk of stroke have not yet been published. This is also true of the previous SARS pandemic that only affected 8000 individuals. Although, an approximate stroke incidence rate of 1 per 42 SARS patients was determined from a small, retrospective single-center analysis.^17^ For now, assumptions on the prevalence of stroke among COVID-19 patients are based on small, single center observational studies,^18^ which estimate an incidence rate of approximately 5% among the most severe cases. In a larger single center study of 3556 COVID-19 patients the estimated stroke incidence rate was much lower at 0.9%.^19^

It is likely that any estimation of stroke incidence will be confounded by under-reporting; both in severe infection with competing risk of mortality and milder infections (and strokes) not presenting to hospital or primary care.

## Common features of COVID-19 pathogenesis and early pathology of ischemic stroke

As we begin to better understand COVID-19 there are clearly aspects of its pathogenesis and disease course that are implicated in the initiation, or in the very early pathophysiology, of ischemic stroke. What follows herein is a detailed summary of the current literature surrounding COVID-19, encompassing the immune and inflammatory responses to infection, thrombotic manifestations and vascular consequences of infection with a focus on possible mechanisms by which these elements may contribute to acute stroke events.

### The immune response to SARS-CoV-2 infection

**Figure 1.**
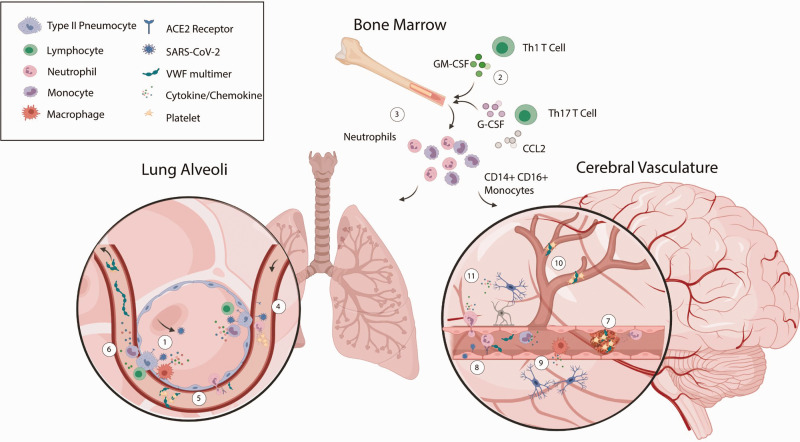
Thromboinflammatory pathways implicated in the pathophysiology of COVID-19 and complicating ischemic stroke. Infection of the lower respiratory tract begins with the binding of SARS-CoV-2 to ACE2 on the surface of type II alveolar pneumocytes (1). The immediate type I interferon response recruits macrophages, monocytes, and neutrophils to the alveoli. Propagation of the innate immune response is directed by Th1 and Th17 CD4^+^ T cells (2) as neutrophils and pro-inflammatory monocytes are targeted to the site of infection (3). Endothelial activation, by either the inflammatory environment or by direct viral infection, upregulates key cell adhesion molecules allowing further infiltration of pro-inflammatory monocytes, cytotoxic T cells and activated neutrophils (4). Endothelial activation also elicits tissue factor release, endovascular recruitment of neutrophils releasing neutrophil extracellular traps (NETs) (4) and von Willebrand factor (VWF) exocytosis from Weibel Palade bodies (5) all of which contribute to the development of microvascular thrombosis. These local immune and pro-coagulant responses may result in systemic release of multiple cytokines and chemokines and ultra large VWF multimers, hyper-activation of circulating platelets and the embolization of VWF/platelet-rich thrombi (6). Increased pro-inflammatory and pro-coagulant factors in the plasma could be sufficient for *in situ* thrombus formation in the cerebral vasculature (7) and this may be exacerbated by infection and/or activation of the cerebral endothelium and local release of VWF and tissue factor (8). Endothelial activation would be expected to facilitate recruitment of neutrophils, monocytes and macrophages to the vessel lumen and induce a local inflammatory response in the surrounding brain parenchyma thereby polarizing microglia (9). Small vessel occlusion, by thromboemboli or by *in situ* thrombosis due to endothelial dysfunction, causes hypoperfusion of brain tissue (10). Ultimately, this combination of tissue hypoperfusion and the pro-inflammatory action of infiltrating and brain resident immune cells is the origin of stroke brain injury (11).

We know, from extensive research on SARS-CoV and other RNA viruses, that initial virus recognition occurs in the ciliated epithelial cells of the upper respiratory tract. This leads to activation of type II alveolar pneumocytes and innate immune cells, culminating in the activation of type I interferon (IFN), production of pro-inflammatory cytokines (interleukin-6 (IL-6), tumor necrosis factor (TNF)) and the induction of IFN-stimulated genes^20^ (Figure 1(1)).

The immediate immunomodulatory impact of type 1 IFN (and the subsequent type 2 IFN (IFN-γ) response) manifests as an accumulation of pro-inflammatory monocytes and macrophages in the alveoli.^21^ Recruited macrophages are themselves prominent sources of IL-6, monocyte chemoattractant protein-1 (CCL2), IL-8 and IFN,^22^ thereby contributing to further influx of myeloid cells (Figure 1(1)), including neutrophils, which are an indispensable component of the inflammatory response to infection but also a major contributor to lung pathology.^23^ Extensive neutrophil extravasation into the alveolar space has been identified in post-mortem tissue of COVID-19 patients^24^ and neutrophil activation has long been associated with disease pathologies involving ARDS, and correlates with the extent of lung damage and with cytokine levels (TNFα, IL-6 and IL-8).^25^

The release of pro-inflammatory cytokines (type I IFN and IL-6) also aids the initiation of the adaptive immune response to viral infection, through the maturation of lung resident conventional or monocyte-derived dendritic cells (DCs).^26^ These DCs migrate to draining lymph nodes and elicit a robust activation and proliferation of naïve, antigen-specific CD8^+^ (“cytotoxic”) T cells and naive CD4^+^ (“helper”) T cells.^27^ In a cohort of 128 SARS patient samples, a high frequency of CD8^+^T cells, Th1 and Th2 CD4^+^T cells and polyfunctional CD4^+^T cells were identified in blood from patients with severe disease.^28^ Early indications from COVID-19 patients suggest a similar clonal expansion of CD4^+^ and CD8^+^ T cells,^29^ and differentiation of Th1^30^ and Th17 cells.^31^

A paradoxical, but common, characteristic of SARS-CoV-2 infection is the development of lymphopenia, observed also in the SARS-CoV, MERS-CoV and H_1_N_1_/H_7_N_9_ influenza pandemics.^32^ In COVID-19 patients, a marked decrease in the number of peripheral CD4^+^ and CD8^+^ T cells, accompanied by hyperactivity of these populations,^31^ has been suggested as an effective predictor of disease severity.^33^ It has been postulated that this may be the result of virus-mediated dysfunction of lymphatic tissues, cytokine-mediated lymphocyte apoptosis or the direct killing of lymphocytes by viral infection.^6^ It could also be, simply, the redistribution of lymphocytes to the infected tissue and/or lymph nodes.

The net result of lymphopenia, particularly in combination with enhanced granulocytosis, is an increase in the neutrophil to lymphocyte ratio (NLR). Increased NLR is already evident in COVID-19 cohorts and has been suggested as a potential prognostic marker of more severe illness and increased risk of mortality.^34^ This is an important observation given that NLR also has prognostic value for determining stroke risk.^35^

### The exaggerated immune response (“The Cytokine Storm”)

The cumulative consequence of leukocyte recruitment and activation is the accumulation of cytokines, both in the lung tissue and in the circulation. In severe COVID-19 patients, this response is exaggerated, resulting in a “cytokine storm”, in which aberrant cytokine expression and disproportionate inflammation results in persistent acute lung injury extending beyond the time of peak viral load. Cell populations of particular relevance to the development of a cytokine storm are pro-inflammatory (CD14^+^, CD16^+^, IL-6^hi^) monocytes and pathogenic (GM-CSF^+^, IFN-γ^+^) Th1 lymphocytes, which appear to predominate in the circulation of COVID-19 patients in ICU,^30^ and Th17 lymphocytes which are prevalent in influenza.^36^ Together these cells propagate a second wave of immune cell infiltration,^30^ polarization of lung-resident macrophages to a pro-inflammatory phenotype^37^ and cytokine production (Figure 1(2) and (3)).

Macrophage polarization rapidly elevates the levels of circulating cytokines, most notably IL-6 (Figure 1(6)). In all of the COVID-19 cohorts included in a recent meta-analysis, IL-6 levels were significantly elevated (approximately 3 fold) in patients with complicated disease compared to those with non-complicated disease.^38^ In the largest of the individual studies (*n* = 452), the median plasma concentration of IL-6 was 25.5 pg/ml,^39^ in line with levels observed in severe SARS-CoV^40^ and MERS-CoV infection.^41^

The exaggeration of peripheral immune responses and ensuing inflammation is likely to be one of the key aspects of COVID-19 pathogenesis that could result in cerebrovascular events. This is highlighted by the observation that IL-6 is a predictor of stroke risk.^42^ It is possible that hyperinflammation may contribute to the progression of two key stroke risk factors, atherosclerosis and atrial fibrillation (AF). In the case of chronic atherosclerosis, viral infection is thought to drive the progression of atheromatous plaques through enhanced macrophage and T-cell responses^43^ within the developing lesion, although this may not occur within a timeframe that is relevant to COVID-19 related stroke. However, the release of IFN-γ, TNF-α and other destabilizing factors can then expose the plaque's thrombogenic core^44^ inducing plaque rupture which is more likely to be influenced by acute inflammatory conditions. There is also some evidence that plaque development and rupture is influenced directly by viral infection of vascular cells.^45^ Similarly, peripheral immune responses to viral infections are thought to contribute to the pathogenesis of AF through the release of reactive oxygen species and myeloperoxidases from neutrophils and the local release of TNF-α and IL-1β from macrophages.^46^ Viral infection may also be an important factor in the development of non-valvular AF through upregulation of monocyte TLR2 and IL-6 release.^47^

### Thrombotic complications of COVID-19

COVID-19 appears to share many aspects of its pathology with previous viral pandemics including the prevalence of thrombotic complications. During the 2009 H_1_N_1_ influenza pandemic, the incidence of overt thrombotic manifestations (deep vein thrombosis (DVT) or pulmonary embolism (PE)) was approximately 6%.^48^ During the 2002–2003 SARS pandemic, the incidence of DVT was estimated to be as high as 20% with a further 11% of patients developing PE.^49^ In a larger SARS cohort, thrombotic abnormalities were identified in over 60% of patients.^50^ Studies of aberrant coagulation in COVID-19 patients are often reporting on small cohorts and conclusions have been conflicting. In a cohort of 183 patients with severe COVID-19, hemostatic abnormalities were associated with fatality, with 70% of non-survivors meeting the criteria for disseminated intravascular coagulation (DIC), including a progressive increase in PT and D-dimer.^51^ However, in a smaller cohort of ICU patients a state of hypercoagulability, not consistent with overt DIC (increased D-dimer without associated bleeding), was reported indicating extensive pulmonary vascular thrombosis.^52^ This is further supported by a small autopsy series which identified thrombotic microangiopathy that was completely restricted to the lungs.^24^

It is, therefore, still unclear if SARS-CoV-2 infection has a direct impact on hemostatic mechanisms or whether the thrombotic manifestations are purely the result of DIC, secondary to systemic inflammation, or sepsis-induced coagulopathy. There is some emerging consensus regarding the rate of incidence of venous thrombosis (in the absence of overt DIC) in COVID-19 patients, and it is extraordinarily high. Klok et al. recorded an incidence of total thrombotic complications of 31% (95%CI 20–41%, *n* = 184), of which 81% were PE.^53^ The incidence of unspecified venous thrombosis in two smaller studies was 25% (*n* = 81)^54^ and 69% (*n* = 26).^55^ In a larger cohort of patients (*n* = 362), the incidence of thromboembolic events was 16.7% in ICU patients and 6.4% across all COVID-19 patients in general wards.^56^ In all of these studies the incidence of VTE is despite all patients having received, at least, a prophylactic dose of anticoagulants.

There is clear evidence, from post-mortem lung pathology, of extensive thrombosis in the alveolar capillaries and small vessels in response to COVID-19 infection.^24^ The composition of these thrombi includes fibrin deposits, platelet aggregates, CD4^+^ cell aggregates, and partially degenerated neutrophils (Figure 1(4)). Fibrin deposition in response to inflammation is initially driven by elevated C-reactive protein (CRP) and inflammatory cytokines.^57,58^ Local tissue factor release, generating thrombin, coupled with elevated plasma concentrations of fibrinogen^59^ results in deposition of fibrin which persists due to the concomitant suppression of fibrinolysis by CRP-mediated release of plasminogen activator inhibitor-1^60^ and thrombin activatable fibrinolysis inhibitor.^61^ Any other pro-coagulant and/or anti-coagulant factors that may be specifically influenced by SARS-CoV-2, thereby further contributing to the hypercoagulable state, are not yet known and, as most are synthesized in the liver, it may be unlikely that infection will alter their transcription. A comprehensive analysis of the plasma concentrations of coagulation factors in patients with COVID-19, or indeed any viral infection, seems to be lacking at present from the literature.

The presence of platelet-containing thrombi in the lungs of COVID-19 patients indicates the involvement of other thromboinflammatory pathways that upregulate endothelial platelet recruitment and aggregation (Figure 1(5)). This is likely to be initiated by an increase in IL-6, IL-8, and TNF-α which stimulate the exocytosis of von Willebrand factor (VWF) from Weibel-Palade bodies.^62^ The VWF strings released unfold under rheological shear forces and capture platelets through the glycoprotein Ib-IX-V complex, a process down-regulated by the protease ADAMTS13. The synthesis of ADAMTS13 is known to be inhibited by IFN-γ, IL-4 and TNF-α and, through an as yet unknown mechanism, IL-6 inhibits ADAMTS13 activity at the endothelium.^62,63^ Therefore, under inflammatory conditions, there is an amplification of VWF-mediated platelet capture. As an acute phase reactant, VWF (particularly ultra large VWF multimers) has long been associated with acute inflammation^64^ and acute viral infection of the respiratory tract.^65,66^ It is also highly likely that VWF/ADAMTS13 imbalance plays an important role in COVID-induced thrombosis with some small studies already identifying VWF antigen and VWF activity in COVID-19 patients as high as 600–800% of the normal range.^67,68^ Notably, an imbalance in the VWF/ADAMTS13 axis is an established risk factor for the incidence of ischemic stroke^69^ and is already implicated in stroke complicating other viral infections.^70^

The formation of platelet-rich thrombi could be further exacerbated by a local hyper-activation of platelets, the consumption of which would account for mild thrombocytopenia observed in many COVID-19 patients and linked to higher rates of mortality.^71^ Platelet activation and aggregation is likely to be induced by the action of locally generated thrombin through PAR1/PAR4 signaling on platelets. The recruitment of platelets from the circulation may also be supplemented by local production of platelets by pulmonary megakaryocytes. These have been observed, in post-mortem lung pathology, actively producing platelets in the alveolar capillaries.^24^ The significance of platelets in COVID-19 pathology is highlighted by the possible therapeutic benefit of anti-platelet therapies.^72^ Importantly, platelet activation and subsequent degranulation may be an early contributor to the exacerbated immune/inflammatory response to SARS-CoV-2 infection through numerous mediators of leukocyte and endothelial function.^73^ Platelet recruitment of leukocytes to the developing thrombus would explain the notable presence of CD4^+^ T cell aggregates^24^ and neutrophil extracellular traps (NETs),^74^ both of which further contribute to both the pro-inflammatory and pro-coagulant environment.

Recently it has become apparent that stroke-causing thrombi are often rich in VWF, platelets, leukocytes and NETs and that this composition is associated with tissue plasminogen activator resistance.^75^ Formation of thrombi with this composition requires the upregulation of multiple thromboinflammatory components, many of which (described above) are influenced by COVID-19. This, together with the incontrovertible role of hyper-coagulation in ischemic stroke pathology,^76^ forms perhaps the strongest argument for a causative link between COVID-19 and stroke.

### Vasculitis and endothelial dysfunction

Emerging evidence suggests that COVID-19 pathology may be considered, at least in part, a vascular disease and that the effects of SARS-CoV-2 on endothelial function may go beyond the release of tissue factor and VWF already described.^77^ ACE2 is expressed in venous and arterial endothelial cells, arterial smooth muscle cells, and pericytes.^4^ A recent post-mortem case series has indicated that SARS-CoV-2 is capable of productively infecting and damaging endothelial cells across multiple tissue beds (Figure 1(4) and (8)), although, the authors concede that these observations are not conclusive.^78^ Hyperactivation of the endothelium during viral infection is known to induce the loss of tight junctions, vessel permeability and, subsequently, pulmonary hemorrhage, and alveolar edema.^79^ In the later stages of COVID-19 progression, as the disease becomes more severe, it is possible that complement activation may also contribute to vasculitis. This mechanism has been observed previously in animal models of coronavirus infection^80^ and pulmonary biopsies from a small number of COVID-19 patients indicate the presence of complement activation.^81^ In addition to inflammation of the vessel wall, complement may contribute to vascular dysfunction through the initiation of microvascular thrombosis. All of these processes could be expected to occur in the endothelium of multiple organs, including the brain, where alterations of the vascular environment are a key contributor to the development of ischemic brain injury.

### Systemic implications of SARS-CoV-2 infection

It is not yet fully understood how systemic SARS-CoV-2 infection, or systemic effects of COVID-19 disease, affects other organs and tissues. In the context of stroke, the possibility of central nervous system (CNS) infiltration by the virus, infection and/or dysfunction of the cerebral vasculature and a systemic hyper-inflammatory and pro-coagulant state are of particular interest.

The possibility of SARS-CoV-2 neurotropism is intriguing. Many other viruses, including coronaviruses, are capable of infecting CNS related cell types including neurons, microglia, astrocytes, and oligodendrocytes. In COVID-19 patients, CNS infection may account for the high incidence of neurological manifestations (as high as 88% of severe cases^82^) which include headaches, nausea, impaired consciousness, acute cerebrovascular disease, and seizures.^83^ Conversely, these manifestations may simply reflect the remote effects of systemic inflammation. A likely route of CNS infiltration is through peripheral nerve terminals, particularly the olfactory bulb. In a humanized mouse model of SARS-CoV-1 infection, nasal inoculation was followed by dissemination of the virus from the olfactory epithelium to the axons of the olfactory bulb, through the pyriform cortex to the brain stem.^84^ This may also explain the widespread incidence of anosmia occurring in COVID-19 cohorts. There is a strong association between acute CNS infection (e.g. meningitis) and the incidence of stroke^85^ thought to result from vasculitis and a related hyper-coagulant state.

In the case of systemic thrombosis, an important distinction yet to be made is whether all thrombotic events are originating in a pro-coagulant micro-environment within the lungs or if there is a systemic pro-coagulant state facilitating *in situ* thrombus formation in the brain or embolism to the brain from elsewhere, such as the peripheral arterial or venous system (e.g. via patent foramen ovale). The latter is certainly possible through the presence of highly reactive ultra-large VWF multimers, hyper-activated platelets and upregulated cell adhesion molecules (through activation of the endothelium), all of which would be expected in the vasculature of the brain (Figure 1(7)). Endothelial dysfunction, particularly vasoconstriction, may occur as a result of direct infection of either endothelial cells or smooth muscle cells (Figure 1(8)) and may enhance the shear-dependent formation of VWF-platelet aggregates in the smaller vessels (Figure 1(10)). Neutrophils, circulating in high numbers and in an activated state, may contribute further to *in situ* thrombus formation in the brain through the formation of NETs^86^ (Figure 1(7)). The majority of strokes being reported in COVID-19 patients are large vessel occlusions,^18^ which is indicative of a thromboembolic source; however, *in situ* thrombosis cannot be ruled out. Given the high incidence of myocardial injury (myocarditis, ischemia, pericarditis, etc.) associated with severe COVID-19 disease/treatment,^87^ secondary stroke in COVID-19 patients may also have a cardioembolic source.

### Is COVID-19 *directly* contributing to stroke incidence?

As approximately 65% of observed strokes in COVID-19 patients are conventionally cryptogenic,^19^ it may be tempting to prematurely assume a relationship; however, it is very important to emphasize that there is no direct evidence for a causal link between COVID-19 and stroke. Standardized case reporting and the application of Bradford Hill criteria will be essential in defining causality.^88^

Thrombotic and cerebrovascular complications are not uncommon in critically ill patients due to systemic inflammation, prolonged immobility, intermittent AF, sedation, mechanical ventilation, and central catheter placement, but can be effectively prevented by prophylactic anti-coagulants.^89^ This is not the case in COVID-19 (and the previous SARS outbreak) and a recent retrospective cohort study has suggested an incidence of stroke 7–8 times higher in patients hospitalized with COVID-19 infection compared with those hospitalized by influenza,^90^ supporting the possibility of a SARS-CoV-2-driven hyper-coagulant state.^17^ Platform trials of anticoagulant or immunomodulatory therapies in hospitalized patients with COVID-19 present a unique opportunity for gaining insights into the causal role of inflammation and thrombosis in stroke risk. However, many of these trials focus on short-term outcomes and it is unclear whether there would be sufficient power to detect differences in stroke events, even if incident stroke was recorded as an *a priori* outcome measure.

Comorbidities that are common to both COVID-19 and stroke (hypertension, diabetes, obesity, etc.) may explain, at least some, coincidence of the two pathologies.^91–93^ Obesity, in particular, is emerging as a prominent risk factor in the development of severe COVID-19 disease and is generally associated with increased incidence and increased severity of respiratory viral infection.^94,95^ This is perhaps unsurprising given the existence of low grade inflammation in obese patients^96^ which undoubtedly contributes to the initiation of ARDS. Notably, the cytokine IL-33 is persistently elevated in obese individuals and is capable of stimulating endothelial cells to release pro-coagulant tissue factor^97^ which may expose them to more severe COVID-19 disease and/or stroke. Of course, not all COVID-19 patients who go on to have strokes will have these comorbidities, and this is especially true of younger patients.^18^ It is in these apparently healthy, young individuals that a causal link between COVID-19 and cerebrovascular complications may be the most logical explanation, particularly in asymptomatic individuals with significant and undiagnosed inflammation.

Up until this point, we have discussed the incidence of stroke complicating COVID-19 only in the context of the most severe and often critical cases as it does appear to be a delayed complication. It could, however, be argued that the strongest case for a causal link would be the incidence of stroke in individuals with sub-clinical SARS-CoV-2 infection, which has been identified in a number of cases.^18^ The true extent of community infection is not known due to a failure, in most countries, to introduce widespread testing and contact tracing, although this is changing rapidly. It has been estimated that in China as many as 86% of cases were undocumented^1^ and this is likely to be echoed in other epicenters of the outbreak and will become apparent when a reliable serological test becomes widely implemented. Asymptomatic or mildly symptomatic individuals are also highly unlikely to undergo any kind of medical examination, especially in light of the imposed lockdown measures. Therefore, we do not yet know the extent of systemic inflammation or the likelihood of aberrant coagulation in these mild or asymptomatic patients and whether or not they are at increased risk of stroke. An interesting study of infected individuals aboard the Diamond Princess cruise ship, one of the earliest clusters outside of China, has given some insight into the extent of asymptomatic infection within an isolated, and comprehensively tested population.^98^ Of the 454 confirmed infections on board, almost half were asymptomatic. Follow-up chest imaging, performed on 76 of these asymptomatic individuals, revealed a 54% incidence of abnormal CT findings (mostly ground-glass opacities). This is not a trivial observation as such abnormalities are indicative of established infection and advanced inflammation. Applied to the general population this could indicate that as many as 25% of all infected individuals may have undetected inflammation and may be at risk of thrombotic complications, including stroke.

A proven, direct link between COVID-19 and stroke may only arise when a vaccine is deployed which then results in a reduced stroke incidence in already at-risk groups, as is thought to be the case with the influenza vaccine.^99^ Recent meta-analyses have shown influenza vaccination is associated with a reduced risk of stroke; however, further prospective studies, particularly large, multicenter randomized controlled trials, are required to definitively show this link. Until then, the mere possibility of a causal link will undoubtedly require some adaptation of current clinical practices to better manage their coincidence. This is likely to include changes to neurovascular imaging protocols, thrombolytic administration, and the application of mechanical thrombectomy. Some of these proposed changes have been outlined in an international panel report published recently in IJS.^100^ A better understanding of the mechanisms underlying a link between COVID-19 and stroke will help to adapt these clinical practices further, particularly in regard to the use of thrombolytic, anti-coagulant, and anti-platelet therapies.

## Summary

The SARS-CoV-2 pandemic is by no means the first viral infection to be linked to an increased incidence of stroke. Research interest in this phenomenon has peaked upon the emergence of past epidemics, giving us some insight into possible mechanisms, but has dissipated as the threat is contained (as was the case with SARS and MERS). This represents a missed opportunity to gain valuable insight on the general link between infection and stroke.

Similarly, we may have missed the first opportunity to study the present pandemic as the number of new cases in the main epicenters of the outbreak begin to decline. Studies of SARS-CoV-2 so far have been generated at a staggering rate, possibly at the expense of scientific rigor. Many have been small, case series studies that could be viewed as less insightful than larger, collated clinical datasets.

It is gradually becoming clear from the early epicenters of the outbreak (e.g., China, South Korea) that SARS-CoV-2 has not yet been fully contained and, with any potential treatments or vaccine still months away, a second wave remains possible. In addition, the implications of any potential causal relationship between SARS-CoV-2 and stroke risk on the primary and secondary prevention of stroke is yet to be determined. Finally, the impact of COVID-19 combined with seasonal influenza on stroke risk remains uncertain. In the meantime, the research community should be preparing to employ large systematic clinical studies and establishing animal models of COVID-19 to confirm the causative mechanisms by which stroke might occur. Only then we will be prepared for the next viral threat and the cerebrovascular risk it may pose.

## Search strategy and selection criteria

Searches were performed in Pubmed using the search terms “COVID-19”, “SARS-CoV-2”, “COVID-19, stroke”, “SARS-CoV-2, stroke”, and “viral infection, stroke”. Searches were performed periodically from 13 April 2020 to 4 June 2020. Search results were screened for relevance by multiple authors. Expedited publications and those on pre-print servers were scrutinized by multiple authors for scientific rigor before inclusion.
